# Soluble and EV-Associated Diagnostic and Prognostic Biomarkers in Knee Osteoarthritis Pathology and Detection

**DOI:** 10.3390/life13020342

**Published:** 2023-01-27

**Authors:** Marko Moravek, Jana Matejova, Timea Spakova

**Affiliations:** Associated Tissue Bank, Faculty of Medicine, Pavol Jozef Safarik University and Louis Pasteur University Hospital, Tr. SNP 1, 040 11 Kosice, Slovakia

**Keywords:** osteoarthritis, knee joint, biomarkers, extracellular vesicles

## Abstract

Osteoarthritis (OA) is the most common degenerative disease of the connective tissue of the human musculoskeletal system. Despite its widespread prevalence, there are many limitations in its diagnosis and treatment. OA diagnosis currently relies on the presence of clinical symptoms, sometimes accompanied by changes in joint X-rays or MRIs. Biomarkers help not only to diagnose early disease progression but also to understand the process of OA in many ways. In this article, we briefly summarize information on articular joints and joint tissues, the pathogenesis of OA and review the literature about biomarkers in the field of OA, specifically inflammatory cytokines/chemokines, proteins, miRNA, and metabolic biomarkers found in the blood, synovial fluid and in extracellular vesicles.

## 1. Introduction

Osteoarthritis (OA) of the knee joint is one of the most widespread musculoskeletal system diseases. OA is accompanied by number of symptoms, such as strong knee joint pain, articular cartilage degradation, synovial membrane inflammation and pathological enlargement of the subchondral bone [1]. OA was considered as an excessive wear and tear disease for a long time. This theory was supported by the fact that cartilage contains only one type of cell (chondrocytes), which does not have a high regenerative capacity. The regenerative process is demanding due to the fact that cartilage is not physiologically innervated or vascularized. Subsequently, the wear and tear theory was substituted by the inflammatory environment theory. The theory is based on the action of molecules such as cytokines, chemokines and prostaglandins. These inflammatory mediators significantly influence the production of matrix metalloproteinases (MMPs) and thereby contribute to the gradual degradation of joint cartilage [2].

Cytokines are secreted by chondrocytes, synovial fibroblasts and various other cell types present in the injured knee joint. Chemokines and other protein and non-protein inflammatory mediators are potential molecular biomarkers of OA. They are detectable in the blood, urine and synovial fluid (SF) of OA patients. It would be possible to predict the early stages of OA by identifying a single biomarker or group of biomarkers, which would lead to the early detection of OA and eventually reverse the disease progression.

These potential biomarkers of early-stage OA are not only detectable in soluble form in bodily fluids, but also in extracellular vesicles (EVs) produced by different cells. EVs are naturally occurring cell-derived particles that are surrounded by a lipid bilayer, are incapable of replicating, and lack a functioning nucleus. They are synthesized by several cell types and are present in various bodily fluids (serum, urine, cerebrospinal fluid and SF). EVs were formerly thought to be byproducts of cell metabolism. Nowadays, studies revealed that they perform a variety of crucial functions. Biologically active molecules found in EVs include proteins, messenger RNA (mRNA), microRNA (miRNA) and long non-coding RNA (lncRNA). EVs are considered to be able to influence the microenvironment and offer intercellular communication because of their unique “cargo”.

In this review, we intend to provide an updated overview of the OA pathogenesis, by emphasizing the currently available data and future perspectives of relevant biomarkers, which might augment the diagnosis, monitoring and prediction of this disease.

## 2. Osteoarthritis—Overall

The high prevalence of OA is the main cause of musculoskeletal system disabilities around the world. The disease affects weight-bearing joints, especially hip and knee joints [3]. It is the most frequent type of joint disease limiting the movement of people. Therefore, OA is associated with high health-care costs [4]. The disease is characterized by degradation of articular cartilage, osteophytes’ formation, subchondral bone sclerosis and synovitis present in the injured joint. These degenerative processes cause the common clinical manifestations of OA as chronic pain, stiffness, joint instability, joint space narrowing and other various deformities regularly detected by X-ray [5]. Diagnosis of OA is possible based on typical symptoms of the disease or radiologically using X-ray, MRI or USG. Pathogenesis of OA includes focal progressive degradation of the hyaline cartilage and structural changes to the subchondral bone followed by osteophyte formation [6]. The synovial membrane is a soft tissue protecting joint capsule and is also significantly affected during the development of OA. Throughout the disease progression, immune cells are transferred from the vascular compartment into the synovium [7].

Many MRI-diagnosed osteoarthritic patients are totally asymptomatic [8]. Typical symptoms of OA combined with the occurrence of pathological processes are the most suitable method to diagnose OA [9].

In the past, OA was associated only with articular cartilage destruction caused by systematic overloading. Nowadays, it is known that synovitis as well as inflammatory mediators are crucial elements in OA pathology. Accordingly, the therapy along with the diagnosis of OA is focused on this molecular level [10].

Two types of OA are known, primary and secondary. Primary OA is a hereditary, or age-related disease. It affects predominantly the distal interphalangeal joints of hands, hips and knees. Secondary OA is characterized as a result of an injury associated with trauma, excessive overloading, or metabolic syndrome [11,12]. Injury, such as knee bending and repetitive stress on a joint, can damage a joint and increase the risk of OA in the joint. It is a complex disease as a result of the combination of systematic and biomechanical factors [13]. Although the incidence of OA increases with age it is not an indispensable effect of ageing. Age is always the strongest factor affecting the pathology of OA. Other factors include sex, biological race, bone density, estrogen level (decreasing significantly in women after menopause), nutrition and heredity. Biomechanical factors include increasingly widespread obesity, muscle weakness, various types of joint injuries and malformations [14]. Hereditary manifestation of the development of the disease does not mean the disease will develop into a pathological condition in the future [15]. Occurrences of hypertension, hypercholesterolemia and increased level of glucose in serum are usually coincidences associated with unilateral and bilateral knee OA. These facts suggest that OA is composed of systemic as well as metabolic factors [16].

### 2.1. Pathophysiology of Osteoarthritis

**Figure 1:**
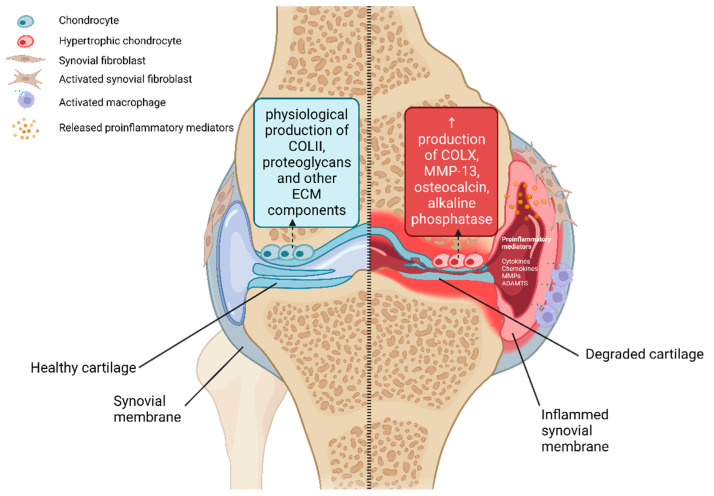
Structural and molecular changes during OA process. Chondrocytes present in healthy articular cartilage (**left**) produce ECM components under physiological conditions. Hypertrophic chondrocytes present in the affected OA joint (**right**) produce degradative enzymes such as MMP13 and the typical hypertrophic chondrocyte marker COLX. Created with BioRender.com.

OA is a complex disease and its pathogenesis is composed of biomechanical as well as metabolic factors. Over time, these factors significantly affect the tissues present in the joint. It is well-known that genetic susceptibility is one of those factors and has an immense impact on the origin and progression of OA [17]. In the healthy synovial joint, the subchondral bone is protected by articular cartilage. Articular cartilage is composed of a dense extracellular matrix (ECM) with a rare distribution of highly specialized cells called chondrocytes. The main functions of that strong connective tissue are to spread the weight of the body and decrease the friction of contact areas during the joint motion. Mechanical damage of articular cartilage causes rapid loss of these functions. Previously mentioned irreversible changes of the cartilage structure cause a joint space narrowing and during end-stage OA, contact of opposite bones is observed. This terminal stage of OA is accompanied by osteophytes’ formation and chronic inflammation in the affected joint. During the inflammation in the joint capsule, a huge number of various cytokines and metalloproteinases are produced into the microenvironment (Figure 1) [14]. These inflammatory-associated molecules support other degradation processes in the affected joint. Studies confirmed that this molecular mechanism is the main cause of the progression of OA [17].

### 2.2. Structural Composition of Articular Cartilage

The components of the ECM of mature articular cartilage are produced by specific cells—chondrocytes [18]. The ECM mainly consists of a dense grouping of collagen fibers (mainly type II, but also IX, XI, etc.), which are stored in a jelly-like environment of proteoglycans [19]. The structural composition of the matrix ensures the strength and elasticity of the cartilage and helping to maintain the correct biomechanical function of the joint [20]. Chondrocytes are the special cells present in cartilage. Their role is to ensure the synthesis of ECM components (collagens, proteoglycans) as well as the synthesis of degradative enzymes (MMPs) during the maturation of articular cartilage [17]. There is a balance between these processes under physiological conditions. In the process of the development of OA, this balance is significantly shifted to the side of degradation processes [21]. Significant structural changes occur in the subchondral bone as part of the body’s reaction to inflammation. These changes are associated with the appearance of sclerosis and the formation of osteophytes, which can be detected by radiographic examination [10]. Changes in the structure of articular cartilage during the process of OA are also associated with phenotypic changes of the chondrocytes themselves [22]. In the initial phase of OA, chondrocyte hyperplasia and subsequent formation of clusters of these specific chondral cells occurs. Superficial chondrocytes synthesize molecules that are released under physiological conditions by hypertrophic chondrocytes located in the lower layer of hyaline cartilage [23]. Hypertrophic (terminally differentiated) chondrocytes are responsible for endochondral ossification, during which bone formation occurs from the original cartilage tissue. Signaling molecules produced by osteoarthritic chondrocytes (pathological state) include osteocalcin, transcription factors c-Maf and Runx2 and the specific marker of hypertrophic chondrocytes collagen type X (Figure 1) [22,24]. Furthermore, the enzyme MMP-13 is produced. This enzyme can efficiently cleave collagen type II and thereby contributes to the destruction of the cartilaginous ECM [22].

### 2.3. Synovial Fluid

SF is a suitable source of information for monitoring the development of the pathogenesis of OA. This type of bodily fluid directly connects and ensures communication between tissues in the joint capsule, such as the synovial membrane, cartilage, infrapatellar fat pad (IFP), etc. Pathological changes in the SF composition are detectable earlier compared to other bodily fluids (e.g., plasma) [25]. Cartilage and SF in healthy joints cooperate with each other to reduce the friction present in the joint during movement. SF provides mechanical shock absorption, lubrication and nutrition of the cartilage. It is produced by the inner membrane of the joint (synovial membrane) and mainly contains serum albumin, hyaluronan, lubricin and γ-globulins. The friction that occurs in the joint during physiological movement changes the composition of the SF. A naturally thin layer of homogeneous fluid is transformed into a thicker, heterogeneous mixture of dense fluid and precipitates due to frictional forces [26]. SF is therefore a typical example of a non-Newtonian fluid.

### 2.4. Synovial Membrane

The synovial membrane is a specialized connective tissue that lines the diarthrodial joints and is responsible for maintaining the volume and composition of the SF. The SF components are mainly produced by cells present in the synovial membrane [7]. These components contribute to the unique functional properties of joint surfaces and modulate the activity of chondrocytes. Lubricin and hyaluronic acid are two main molecules produced by synovial membrane cells. These molecules protect and maintain the integrity of the articular cartilage surfaces in diarthrodial joints. The synovial membrane consists of two layers. The outer layer (subintima) is up to 5 mm thick and consists of several types of connective tissues. This layer contains predominantly type I collagen and is vascularized and predominantly acellular. The inner layer (intima) is in direct contact with the joint cavity and consists of a layer of cells with a thickness of 20–40 μm. These synovial cells called synoviocytes consist mainly of fibroblasts and macrophages. Fibroblasts are the dominant cell population in the healthy synovial membrane [27]. During disease progression, the synovial membrane is a source of proinflammatory and catabolic mediators (MMPs, aggrecan, etc.) [28]. The articular cartilage is not independently vascularized. It is dependent on the neighboring tissues (synovial membrane, subchondral bone) which provide it with the nutrients necessary to maintain vitality [29]. The synovial membrane is semipermeable, i.e., it is responsible for the transfer of nutrients and waste products within the joint space. The synovial membrane is also a rich source of multipotent mesenchymal stem cells (MSCs). These cells are capable of differentiating into multiple tissue-specific lineages including cartilage, bone, muscle and adipose tissue [30]. Therefore, the synovial membrane is a suitable candidate as a source of cells and their products, which can participate in the regeneration of affected components of joint connective tissue [31].

### 2.5. Synovial Inflammation

The role of inflammation in the process of OA was unclear and often debated for a long time [32]. OA was not officially classified as an inflammatory disease based on the number of leukocytes present in the SF. Only articular cartilage degradation and pathological bone growth were considered as features of OA [33]. Furthermore, much higher levels of pro-inflammatory serum biomarkers and more pronounced synovial inflammation (synovitis) were found in inflammatory joint diseases such as rheumatoid arthritis (RA) compared to OA [34]. This finding contributed to the use of alternative names for the disease, such as osteoarthrosis or degenerative joint disease [35]. Increasingly, OA was associated only with mechanical damage to joint components without the direct influence of chronic inflammation [36]. Studies testing anti-inflammatory therapies that failed to modify the progression of OA confirmed that OA belongs to the group of non-inflammatory diseases [37]. However, modern laboratory techniques allowing extensive analyzes of cellular, molecular and genetic factors associated with OA showed that OA is also associated with inflammatory processes. Studies established constant low-grade inflammation and activation of innate inflammatory pathways as mediators of OA pathogenesis [2]. Synovial inflammation is a major factor associated with the risk of cartilage degradation and disease symptoms, including joint pain, swelling and stiffness. Synovitis is commonly present in OA joints. The synovitis in OA is macrophage-predominant, whereas RA synovitis is T-cell-predominant. This reflects the activation of the innate immune response in OA joints, possibly caused by damage to joint tissues that have resulted from a chronic wound in the environment. OA synovitis is more focal than in RA; in the knee, it is commonly located in the suprapatellar pouch. Synovitis plays a significant role in joint destruction in RA, while its role in OA progression may be limited to a subset of individuals. Synovitis involving infiltration of mononuclear cells into the synovial membrane and production of pro-inflammatory mediators is common in all stages of the disease [38]. Histologically, the synovial membrane of patients with OA is characterized by hyperplasia, fibrosis and increased vascularization [39]. Infiltration of leukocytes from the vascular compartment occurs in response to cytokines and cell adhesion molecules [29]. Studies confirmed that macrophages and T-lymphocytes are the most abundant immune cells in the affected synovium. Mast cells, B cells and plasma cells are present in the synovial membrane to a significantly lesser extent [40].

## 3. Potential Soluble Biomarkers of Osteoarthritis

Currently, the evaluation of OA is still based on radiological examinations and subjective pain assessment. Therefore, there is an effort to discover a molecular biomarker of early-stage OA. A biomarker is a characteristic that is objectively measured and evaluated as an indicator of normal biological process, pathogenic processes or pharmacological responses to a therapeutic intervention [41]. To identify early-stage OA predictors, biochemical indicators are now being investigated as the most promising disease markers [42].

Molecular biomarkers are typically measured in bodily fluids such as serum, plasma, urine or SF. Since OA develops inside the knee joint, SF is likely to be the most suitable source of information to detect early-stage OA. Engaging in the effort to detect the early-stage OA provides an opportunity to initiate appropriate therapy. The most plausible options for OA biomarkers are structural molecules or fragments associated to cartilage, bone or synovium. They may be unique to a particular kind of joint tissue or present in all joints [43]. The potential candidates for OA biomarkers discussed in this review are shown in Table 1.

### 3.1. Inflammatory Biomarkers

#### 3.1.1. Cytokines and Chemokines

Previous studies confirmed the positive relation between the progressive degradation of articular cartilage and the presence of synovial inflammation [75,76,77]. Therefore, the production of pro-inflammatory cytokines/chemokines in the process of OA significantly contributes to the catabolic processes occurring in the joint capsule.

The main pro-inflammatory cytokines in the pathogenesis of OA are interleukin-1β (IL-1β)—associated with cartilage degradation, tumor necrosis factor α (TNFα)—controlling the cascade of inflammatory processes and IL-6—one of the main regulatory cytokines in inflammatory reactions in the body. IL-1β and TNFα are produced by chondrocytes, osteoblasts, synovial fibroblasts and mononuclear cells (lymphocytes, monocytes) [78]. These cytokines regulate the production of other pro-inflammatory and catabolic factors. Elevated levels of IL-1β and TNFα were confirmed in SF, synovial membrane, subchondral bone and also in cartilage in patients with OA [79].

Cytokine IL-1β can activate cells through a specific IL-1RI receptor (CD121a) located on the cell surface. Molnar et al. confirmed that expression of the IL-1RI receptor on the surface of OA chondrocytes and synovial fibroblasts was increased in vitro [80]. The binding of IL-1β to the receptor IL-1RI from the Toll-like receptor (TLR) family causes a multistep activation of transcription factors. It leads to increased gene expression and results in the production of other cytokines, chemokines, adhesion molecules and various degradation enzymes [81]. IL-1β significantly contributes to the composition of the ECM by influencing cell metabolism. It reduces the production of the two main ECM components (collagen type II and aggrecan) directly in the chondrocytes in vitro [82]. IL-1β also affects the synthesis of MMPs (MMP-1, interstitial collagenase; MMP-2, gelatinase A; MMP-3, stromelysin-1; MMP-8, neutrophil collagenase; MMP-9, gelatinase B; MMP -13, collagenase 3; MMP-14 or MMP-16) capable of degrading the cartilage ECM [83].

Together with IL-1β, TNFα is considered the main pro-inflammatory cytokine influencing the pathogenesis of OA. TNFα is one of the 19 ligands of the tumor necrosis factor superfamily and binds to two specific receptors (TNFRI and TNFRII) located on the cell membrane [84]. In the same way as IL-1β receptors, TNFα receptors are overexpressed on the surface of chondrocytes and synovial fibroblasts in OA [79]. TNFα also negatively affects the production of proteoglycan components and collagen type II by the chondrocytes [85]. TNFα-activated chondrocytes produce MMP-1, MMP-3, MMP-13 and disintegrin and metalloproteases with thrombospondin motifs (ADAMTSs) responsible for the breakdown of aggrecan [86]. TNFα activity usually corresponds with IL-1β action, and there is a notable synergism between the two cytokines in the case of many phenomena that occur throughout OA. Furthermore, IL-1β and TNFα stimulate the production of inducible nitric oxide synthase (iNOS), cyclooxygenase-2 (COX-2) and prostaglandin E2 (PGE2) synthase, increasing the quantities of their products [87]. Therefore, one of the potential therapies for OA is focused on reducing the negative effect of IL-1β and TNFα [79]. Faster senescence and more frequent apoptotic processes are shown by chondrocytes that are affected by IL-1β and TNFα [88,89,90].

Another main regulatory cytokine in inflammatory reactions in the body is IL-6 that can activate the immune system and strengthen the body’s immune response in a wide range of inflammatory conditions. The production of IL-6 in affected tissues is the result of the action of IL-1β and TNFα. IL-6 is produced in the injured joint by chondrocytes, osteoblasts, synovial fibroblasts, macrophages and adipocytes [91]. Increased levels of IL-6 were observed in the SF and the serum of OA patients. These levels correlate with the radiographically confirmed severity of OA [47]. IL-6 regulates changes in the subchondral bone and supports the formation of osteoclasts and thus the resorption of bone tissue [92]. IL-6 in cooperation with IL-1β and TNFα reduce collagen-type II production and contributes to the overproduction of MMPs [93].

These cytokines are activators of a large number of signaling pathways that activate the production of other inflammatory mediators. This cascade also includes chemokines that can attract inflammatory cells to the affected microenvironment and thereby support the secretion of other inflammatory factors and the progression of the disease [80]. The proliferation, differentiation and activation of cellular responses are all impacted by their diverse range of activity [94]. In OA, chemokines act as mediators of dysregulated joint-tissue metabolism. Based on the position of the N-terminal cysteine residues, these small (8–12 kDa) protein ligands are grouped into four families: C, CC, CXC and CX3C. The great majority of chemokines are members of the CC and CXC families [80].

The most significant CC family chemokines that are associated with OA are CCL2 (monocyte chemoattractant protein-1, MCP-1), CCL3 (macrophage inflammatory protein-1 alpha, MIP-1α), CCL4 (macrophage inflammatory protein-1 beta, MIP-1β) and CCL5 (Regulated upon Activation, Normal T cell Expressed and Secreted (RANTES)) [94]. CC chemokines have a key role in macrophage recruitment and are increased following joint damage in OA.

CCL2 is a ligand of C-C-chemokines-receptor-2 (CCR2) that is expressed in chondrocytes, osteoblasts and synovial cells and has a function in bone metabolism and OA [78]. CCL2 functions by aggregating monocytes in the host defense and macrophages at the site of inflammation. CCL2 expression was elevated in individuals with a variety of inflammatory diseases, including OA. CCL3//MIP-1 α is a chemotactic chemokine released by macrophages. It carries out a number of biological processes, including the recruitment of inflammatory cells, wound healing, the suppression of stem cells and the maintenance of effector immune responses. Furthermore, CCL3 stimulates osteoclastogenesis, which is important in the bone remodeling process and CCL3-expressing cells are typically found in sites of inflammation and bone resorption [95]. CCL4 is a small cytokine of the CC chemokine family. CCL4 is released in response to mitogenic signals and antigens, acting as a chemoattractant for natural killer cells, monocytes and other immune cells at the site of inflamed or injured tissue [96]. CCL4 is regulated by the NF-ΚB signaling pathway. Due to its stimulation of OA chondrocyte apoptosis, studies have shown that the NF-ΚB signaling pathway is strongly associated with OA. The NF-ΚB signaling pathway’s inactivation has been shown to be a helpful factor in the OA process of cartilage breakdown [97]. CCL5 was discovered to be a product of activated T cells and plays an important function in the inflammatory response. CCL5 has been previously linked to chronic inflammatory disorders such as RA, inflammatory bowel disease and cancer. It attracts monocytes, eosinophils and T lymphocytes while also activating eosinophils and basophils to release granule content. It has been shown that chondrocytes expressed CCL5 together with other various chemokines during OA pathological processes [98]. CXCL12 is a CXC family chemokine that is also important in the pathophysiology of OA. By interacting with a single receptor, CXCR4, CXCL12 mobilizes MSCs to sites of injury and therefore could be a potential diagnostic as well as therapeutical tool associated with OA [99].

##### Potential Cytokine/Chemokine Biomarkers in Synovial Fluid

OA is an expensive and debilitating disease that is frequently not detected early enough to stop its progressing. The study of SF biomarkers can advance understanding in the field of knee OA diagnosis and therapy [100]. SF is a suitable source of potential biomarkers because it is in direct contact with the cartilage and synovial layer of the injured joint. OA causes a malfunction in the crucial ECM alterations’ process, which results in a significant loss of cartilage [101]. As a result of aberrant matrix turnover and altered SF composition in an OA-affected joint, numerous molecules and fragments of matrix components from tissues such as articular cartilage, bone and synovium are released into the SF. Some of these fragments can be measured in SF even before they can be found in other samples such as serum and urine [102]. Therefore, SF can more accurately represent the changes caused by OA in a specific joint. Since a pro-inflammatory environment can negatively impact the health of chondrocytes and the maintenance of a healthy ECM, local inflammatory cytokines such as IL-1β, TNF-α and IL-6 are crucial in the process of OA formation and development [103]. Additionally, there is rising evidence that the pathophysiology of OA is influenced by the local production of pro-inflammatory cytokines and chemokines [87]. Chondrocytes, osteoblasts, synovial cells and mononuclear cells present in the microenvironment of the affected joint are able to produce a large number of these inflammatory mediators. Therefore, SF is a strong candidate for discovering important OA biomarkers.

Monibi et al. used a multiplex test to examine SF samples from 18 individuals. When compared to healthy individuals, MMP-1, IL-6, IL-8 and CCL5 were all significantly higher in the SF of OA patients. They also confirmed that CCL2 was strongly linked with IL-6, IL-8 and the radiographic OA severity ratings further support the idea that it is a useful biomarker for clinical diagnosis [48]. Li et al. confirmed a positive correlation between CCL2 concentrations in SF and the Western Ontario and McMaster Universities index (WOMAC) pain, function, and overall scores [104]. CCL2 and CCL5 can stimulate MMP-3 expression in vitro, which causes proteoglycan loss and cartilage degradation in the OA inflammatory microenvironment [105]. Chemokine expression in SF, synovium and cartilage was examined in patients with and without knee OA [106]. In comparison to controls, the SF of knee OA patients had higher levels of CCL2, CCL3 and CCL4. Inflammatory mediators in the SF that are connected to nociceptive and neuropathic pain in knee OA were analyzed by Li et al. [107]. They found that expression of IL-1β and IL-6 was increased in the early-stage OA group compared with the late-stage OA group. The WOMAC pain score has no correlation with any of the inflammatory mediators analyzed. Beekhuizen et al. analyzed the levels of 47 mediators in the SF of control donors and OA patients [50]. The majority of the mediators were found in both the control and OA SF samples. Some of them, such as IL-6 and CCL5, were shown to be significantly higher in OA compared to controls. CCR2 ligands (CCL2, CCL7, CCL8) were found to be increased in SF from human OA and post-traumatic knees [108]. CCR2-expressing cells were found in higher numbers in OA synovium, and CCR2-expressing macrophages were detected by immunofluorescence at areas of damage, suggesting that this pathway is also involved.

There is evidence that articular chondrocytes express CXCR4, and CXCL12 also stimulates MMP-13 and several other catabolic mediators in addition to its effect on MSCs. The role of CXCL12/CXCR4 signaling in fracture healing and bone remodeling has been established [109]. CXCL12 levels in SF were shown to be strongly correlated with the radiographic severity of knee OA in a study that included 252 patients with the condition and 144 healthy controls [52]. The Kellgren–Lawrence (K-L) grading method was used for the radiological grading of OA in the knee. CXCL12 levels in the SF of knee OA patients with K-L grade 4 were considerably higher than those with K-L grades 2 and 3. Furthermore, individuals with knee OA with K-L grade 3 showed substantially greater SF levels of CXCL12 than those with K-L grade 2.

In-depth research in this field has been limited since many patients with degenerative OA have small volumes of SF in the intra-articular space [78]. Although SF only reaches small volumes, it is nevertheless highly likely to be the best source of OA biomarkers.

##### Potential Cytokine/Chemokine Biomarkers in Blood

Since peripheral blood contains tens of thousands of different proteins and many of them are produced by numerous extra-articular sites, measuring specific cytokine, chemokine or MMP levels in the peripheral blood may not accurately reflect levels in the synovial space. Blood is considered a less specific source of information when searching for a suitable OA biomarker compared to SF. Research also focuses on the study of blood because it is a much easier source to obtain from the patient. Many studies are therefore focused on the research of such biomarkers that could reveal the progression of OA in the blood.

Independent of other risk variables, the severity and development of symptomatic knee OA were marginally and causally correlated with plasma levels of IL-1 receptor antagonist (IL-1Ra) [110]. These findings are encouraging for the identification of predictive biomarkers involved in IL-1 signaling as well as the development of innovative drugs for the treatment of OA. According to Runhaar et al., variations in serum levels of IL-6, TNF-α, IL-1R and CRP throughout the intervention period explained 15% of the change in WOMAC pain ratings and 29% of the change in WOMAC function scores [111]. These findings emphasize the importance of changes in systemic inflammation as drivers of clinically significant effects following diet and exercise in overweight and obese people with knee OA. Nordahl et al. confirmed that the presence of IL-1β in plasma and SF is associated with temporomandibular joint (TMJ) radiographic alterations. The extent of erosion and the severity of radiographic alterations to the TMJ were both larger in individuals with detectable IL-1β levels in their plasma than in those without [44]. Panina et al. revealed that early post-traumatic OA was associated with the circulating level of IL-1β in serum and SF [45]. Before routine radiography, combined serum IL-6, TNF-α, and leptin levels can be used as biomarkers to differentiate between post-traumatic OA patients and healthy controls [112]. Combined levels of these three potential biomarkers positively correlated with the K-L score of post-traumatic OA patients. The peripheral blood of OA patients has higher levels of CCL2 and CCL3, which have great predictive value for the incidence, effectiveness and prognosis of recurrence of OA, suggesting their potential roles as excellent markers for OA diagnosis and therapy in the future [113]. According to the study by Zhao et al., CCL3 may be a serum biomarker for knee OA with the ability to identify alterations that are not yet visible on X-rays and classify the severity of knee injury [49]. A total of 181 subjects were included and subdivided into three subgroups (control subjects, pre-X-ray-defined knee degeneration patients and X-ray-confirmed knee OA patients). In the pre-X-ray-defined knee degeneration patients, articular cartilage loss was measured during arthroscopy using the International Cartilage Repair Society (ICRS) classification or recorded on MRI with chondral whole-organ magnetic resonance imaging score (WORMS). The radiographic severity of OA was significantly correlated with CCL13 levels in serum and SF in another study [51]. When compared to knee OA patients with K-L grades 2 and 3, those with K-L grade 4 had substantially higher serum and SF levels of CCL13.

However, many more studies comparing the level of potential cytokine/chemokine biomarkers in SF and blood will have to be carried out to create a standardized protocol for evaluating the appropriate OA biomarker.

### 3.2. Collagenous Biomarkers

#### 3.2.1. C-Terminal Telopeptide of Collagen Type II

Type II collagen is the major component of the articular cartilage matrix [114]. Fragments of C-terminal telopeptide are released following the degradation of type II collagen. At the most common sites of radiographic OA, levels of urinary C-terminal telopeptide of collagen type II (uCTX-II) were shown to be correlated with the overall radiographic score as determined by the K-L method. It was also shown that scores at the knee, hip, facet and hand joints independently contributed to this association [53]. A significant reduction in hand pain and function in individuals with symptomatic hand OA throughout a six-month clinical study with chondroitin sulfate was not connected to a change in uCTX-II levels [115]. uCTX-II correlates strongly with early indicators of inflammatory arthritis, such as bone-mineral density loss and power Doppler ultrasonography synovitis [116]. Following six and twelve months of therapy with glucosamine and chondroitin sulfate or glucosamine, chondroitin sulfate and collagen type II, Scarpellini et al. discovered considerably reduced levels of uCTX-II [117]. Visual analogue scale (VAS) and uCTX-II mean values were considerably lower than the baseline after 6 months and 1 year of treatment. The study consisted of 129 participants (78 in the OA group and 51 in the control group) showed significantly higher levels of uCTX-II in the OA group compared to the control group [54]. Furthermore, uCTX-II levels were independently correlated with the radiographic severity of OA of the knee. The uCTX-II levels and the WOMAC index showed a positive correlation. A comparative study of uCTX-II for knee OA patients and healthy individuals revealed that late-stage OA is associated with higher levels of uCTX-II [118]. Eighty-two patients with knee OA and 20 healthy volunteers were enrolled in this study. Anteroposterior and lateral position X-rays of knee joints were collected. The images were classified using the K-L radiographic grading criteria. The molecular biomarkers CTX-II and IL-1β, which were both considerably elevated throughout the development of knee OA, can be utilized to help with early diagnosis and knee OA therapy [119]. Valdes et al. confirmed in their large-scale meta-analysis the importance of uCTX-II as a degradation product that correlates with a wide range of OA features, including hip, knee and hand OA as well as knee OA progression and radiographic severity of knee OA [55]. In several OA animal models, uCTX-II and serum CTX-II both showed promise for diagnostic and staging purposes. Significant differences in the serum CTX-II levels in rabbits were observed between the adult OA (OA induced by anterior cruciate ligament transection) and the control (unoperated) groups [120]. Csifó et al. studied the effect of meloxicam on knee cartilage degradation in an iodoacetate-induced rat OA model. Within four weeks of therapy, the high-dose group of meloxicam treatment resulted in a remarkable decrease in CTX-II compared to the low-dose and placebo groups [121]. These results suggest an interest in serum CTX-II monitoring for OA progression. Innovative techniques also propose detecting CTX-II in serum and urine simultaneously using a fluoro-microbeads guiding chip [122]. Patients with primary OA had greater concentrations of CTX-II in SF than in the reference group. The mean levels of CTX-II in SF increased above reference values following joint damage at all time points, with the greatest levels occurring shortly after the trauma [123]. Sofat et al. conducted a cross-sectional study on patients with knee OA and healthy controls. A total of 130 patients were evaluated, including 78 with advanced OA who required total knee replacement (TKR), 42 with moderate OA who received conventional therapy and 6 non-OA controls, with four drop-outs. They discovered that increased MRI-detected joint damage was related to higher levels of CTX-II, indicating that MRI and CTX-II biomarkers can be used to monitor OA disease development.

#### 3.2.2. Type II Collagen-Specific Biomarker

Type II collagen-specific biomarker (Coll 2-1) may be described as a biomarker useful for examining the burden of disease, the prognosis and diagnosis based on the Burden of Disease, Investigative, Prognostic, Efficacy of Intervention and Diagnostic (BIPED) categorization system for OA biomarkers published by Bauer et al. [124]. According to research by Henrotin et al., a rise in urine levels of Coll 2-1 or its nitrated form (Coll 2-1NO_2_) over a year was a reliable indicator of the course of joint space narrowing in OA patients [56]. The blood levels of Coll 2-1, a biomarker particular to type II collagen breakdown, can be decreased in knee OA patients by intra-articular injection of reticulated hyaluronic acid with mannitol, according to a randomized double-blind placebo controlled trial. Eligible participants were men and women between the ages of 45 and 80 who had unilateral symptomatic femoro-tibial knee OA that fulfilled clinical and radiologic American College of Rheumatology (ACR) criteria. OA must have been present for more than 6 months, with a mean global knee pain measured on a VAS over the past 24 h above 40 mm (without any analgesics for at least 48 h) [125]. One-year follow-up of Coll 2-1 serum levels in OA patients after hip or knee replacement indicates that Coll 2-1 is a disease-specific marker that is sensitive to the structural changes occurring in a single joint. Additionally, the immunohistochemical results support the idea that injured articular cartilage is the primary source of serum Coll 2-1 [126].

#### 3.2.3. Type II Collagen Cleavage Product

One of the potential biomarkers of early-stage OA is type II collagen cleavage product (C2C). Poole et al. attempted to assess the relationship between early and late knee cartilage pathology and the evolution of cartilage damage using the C2C human urine sandwich assay (IB-C2C-HUSA), a test for cartilage collagenase-mediated degradation. In a population-based cohort of early pre-radiographic disease and radiographic OA, they showed substantial relationships between the novel urine IB-C2C-HUSA immunoassay with cartilage damage cross-sectionally and with cartilage loss longitudinally [57]. To investigate if SF biomarker concentrations corresponded with the severity of radiographic OA and were higher in joints with radiographic OA compared to controls, Coppelman et al. examined SF biomarker concentrations from distal intertarsal and tarsometatarsal joints in adult horses. They confirmed that overall radiographic scores have a positive correlation with the C2C value concentration in equine SF [127]. In addition to the correlations identified with several other injury-related biomarkers, the higher levels of C2C in SF following injury show that an acute knee injury is linked to the rapid and long-lasting local breakdown of type II collagen [128]. These results suggest that C2C could be a suitable OA biomarker.

### 3.3. Non-Collagenous Biomarkers

#### 3.3.1. Cartilage Oligomeric Matrix Protein

A non-collagenous ECM glycoprotein called cartilage oligomeric matrix protein (COMP) is structurally linked to thrombospondins. Initially, COMP was believed to be the unique protein of cartilage, but further research showed that it may also be found in tendons, ligaments and menisci [129]. The cartilage breakdown in OA is measured using the biomarkers COMP and uCTX-II. Increased concentrations of these biomarkers can reveal OA’s severity and prognosis [43]. It was hypothesized that a decrease in both biomarker levels reflects a cartilage recovery. According to a thorough review of OA biomarkers, the most accurate indicators of OA nowadays are COMP, osteocalcin and CTX-II [130]. COMP fragments are released into the SF during the breakdown of articular cartilage. They were discovered in elevated levels immediately following the injury and in the early stages of OA. Plsikova et al. studied SF samples aspirated from the knees of 65 OA patients (46 patients with early-stage OA and 19 patients with end-stage OA according to the K-L grading scale). When compared to the group of patients with end-stage OA, the concentration of COMP in SF was significantly lower in the early OA group of patients. Additionally, the age of the patients and the levels of COMP in the SF were shown to be significantly correlated [58]. Only the baseline level of COMP of the investigated biomarkers was able to predict later MRI-determined cartilage loss in the OA knees [131]. In this study, the WORMS semiquantitative grading method was used to assess baseline and follow-up knee MRI images for cartilage loss. According to the study of Verma et al., early on in the development of knee OA, COMP concentrations in primary OA serum samples were shown to be higher than healthy donors’ values [132]. On a sample of 60 male Sprague-Dawley rats, a study was conducted to evaluate the diagnostic capability of the biomarkers COMP and chondroitin sulfate epitope 846 (CS846). Serum levels of COMP (sCOMP) and CS846 in the model group were considerably greater than those in the control group 10 weeks following surgery [133]. In patients with unilateral hip OA, a group led by Endres attempted to investigate the impact of total hip replacement on sCOMP and its relationship to joint stress during gait [134]. Unexpectedly, the excision of an OA joint had no long-term impact on sCOMP’s level. Independently of age and BMI, high sCOMP levels were linked to an increased risk of incident knee OA, while synovitis was demonstrated to have the biggest impact on COMP levels in established knee OA [59].

#### 3.3.2. S100 Proteins

S100A9 belongs to the alarmin family. It is a crucial protein in the breakdown of cartilage and synovial inflammation in OA. S100A9 expression in cartilage is only seen in hypertrophic chondrocytes and is crucial for the matrix mineralization [135]. The Cohort Hip and Cohort Knee study’s analysis of arthroscopic synovial biopsy samples from patients with early symptoms of OA also uncovered elevated levels of S100A9, which significantly correlated with synovial lining thickness, cellularity in the subintima and joint degeneration [136]. Serum levels of S100A8 or S100A9 were shown to be linked with overall WOMAC scores, weight-bearing pain and physical disability in a study of 141 individuals with clinical knee OA [60].

#### 3.3.3. C-Terminal End-Product of Vitronectin and C3f Peptide

As a ligand for the αVβ3 integrin receptor, vitronectin is degraded by a number of metalloproteinases, including MMP-1,-2,-3,-7 and -9. Vitronectin is a cell adhesion and spreading factor that is highly expressed by bone-resorbing osteoclasts. The C3f peptide is highly expressed, especially in severe forms (K-L 3 and 4). It is also seen in SF, and has a strong correlation with the C-terminal end-product of vitronectin (V65) [137]. De Seny et al. examined 284 serum samples from patients with knee OA from the Bristol OA 500 cohort and the Bristol Validation Study, together with healthy controls and individuals with RA [138]. They discovered four new biomarkers in the serum of OA patients using the SELDI-TOF MS proteomics approach: V65, C3f peptide, the 3762 protein and CTAPIII (Connective tissue-activating peptide III). When compared to controls or those with RA, V65 showed higher amounts in MS spectra in all K-L grades. These findings emphasize its specificity for OA. The C3f peptide is abundantly expressed, particularly in severe instances (K-L 3 and 4), is also seen in SF and has a strong correlation with V65 [137]. Two immunoassays have been created by Ourradi et al. to measure the biomarkers C3f and V65 peptides that were found by their prior proteomic study [139]. These assays were able to identify endogenous peptides in patient and control blood samples, but they lacked the sensitivity necessary to assess peptide levels accurately in patients.

## 4. Potential EV-Associated Biomarkers of Osteoarthritis

Potential biomarkers are not only found in bodily fluids as freely detectable molecules but are also widely present in EVs (EV-associated) [61]. EVs are membrane vesicles with diameters ranging from 30–5000 nm that are released by distinct cells and communicate with one another via paracrine signaling. Exosomes, microvesicles and apoptotic bodies are subtypes of EVs, and research on these EVs has grown dramatically in recent years. Apoptotic bodies are the largest EVs, with sizes ranging from 1000 to 5000 nm. The size of microvesicles ranges from 100 to 1000 nm and exosomes are vesicles typically defined by diameters of 30–150 nm [140]. According to Minimal information for studies of extracellular vesicles (MISEV), EVs should be named either in accordance with size (small EVs <200 nm, large EVs >200 nm), density (low, medium, high), biochemical composition (expression of surface markers) or the origin of the cells from which they originate (e.g., mesenchymal stem cell-derived EVs) [141]. EVs contain and protect useful biological information, including proteins, lipids, lncRNA, mRNAs, and regulatory miRNA [140]. Janockova et al. demonstrated that small EVs and their content can be taken-up by different types of cells isolated from tissues associated with OA (SF, osteoblasts and periosteum-derived MSCs) suggesting their prospective role in the treatment of OA [142]. It is thought that EVs have the ability to act as paracrine effectors and mediators of cell-to-cell communication [62]. Through their functions in intercellular transport, EVs are considered to support a variety of crucial physiological processes including immunological responses, tissue healing and neural communication [143]. EVs show therefore a promising role in tissue regeneration, especially MSCs- and platelet-derived EVs as listed in recently published articles [144,145]. It is difficult to distinguish between early and advanced OA, therefore it is important to investigate and identify sensitive and objective molecular markers of early disease.

### 4.1. EV-Associated Protein/Lipid Biomarkers

The amounts of EV-associated cytokines IL-1β, IL-17, IL-10 and INF-γ found in the SF of patients with late-stage OA were significantly higher than those found in patients with early-stage OA [61]. Zhang et al. identified plasma EVs in OA that carry the major pro-inflammatory cytokines, TNFα, IL-1β and IL-6, demonstrating their pro-inflammatory phenotype [62]. They found that the concentration of TNFα within EVs and the integrated mean fluorescence intensity of TNFα in EVs in OA plasma were associated with and strong predictors of radiographic knee OA progression, while plasma TNFα outside EVs was neither associated with nor predictive of radiographic knee OA progression. Chondrocytes may form exosome-like vesicles (ELV) that are released from cartilage during the matrix loss and cartilage degradation. These ELV may be substantially concentrated in synovial tissue and could have the ability to enter macrophages. The ELV could transfer miR-449a-5p into macrophages and reduce ATG4B expression in lipopolysaccharide-primed macrophages, resulting in autophagy inhibition. Reduced autophagy encourages the production of mitochondrial reactive oxygen species, which increases inflammasome activation and mature IL-1β production. Finally, an increase in IL-1β aggravates synovial inflammation and accelerates the progression of OA [146]. SF is a suitable source of information in monitoring the pathogenesis of OA, because it directly connects the tissues present in the joint capsule, such as the synovial membrane, cartilage or IFP [7]. Based on mass spectrometry protein profiling, Kolhe et al. identified multiple gender (male and female)-specific differential proteins in OA and non-OA EVs. They found that haptoglobin, orosomucoid, and ceruloplasmin were significantly upregulated, whereas apolipoprotein was downregulated in female OA EVs. In males, they discovered β-2-glycoprotein and complement component 5 significantly upregulated and SAGA-associated factor 29 downregulated in male OA EVs [63].

EV-associated lipids could also be a potential biomarker of OA. In the most recent study of Ben-Trad et al. an important finding was reported [64]. They compared healthy and OA samples and showed that the phospholipid (PL) amount increased in pathological samples. However, the PL/neutral lipid ratio decreased, suggesting destabilization of bilayer structures.

### 4.2. EV-Associated miRNA Biomarkers

The 18–25 nucleotide RNA molecules known as miRNAs can control protein translation by complementarily binding to mRNA transcripts [147]. EV-associated miRNA-92a is also involved in mitigating the negative effects of OA by increasing chondrocyte proliferation and ECM synthesis through the PI3K/AKT/mTOR pathway. EV-associated miR-95-5p was shown to enhance chondrogenesis and prevent the development of OA by directly targeting histone deacetylase (HDAC)-2/8. MiR-95-5p suppressed the expression of HDAC2/8 and promoted the formation of cartilage ECM. Therefore, EV-associated miR-95-5p may act as an inhibitor of HDAC2/8, making it a potential diagnostic biomarker of OA [65]. MiR-193b-3p affects histone deacetylase 3 (HDAC3), which in turn affects chondrogenesis and chondrocyte metabolism. Overexpression of this miRNA has been shown to significantly increase cartilage tissue formation in vivo. EV-associated miR-193b-3p plasma levels in patients with OA were shown to be lower than in control samples, thus supporting the positive effect [66].

The WNT5A protein plays a key role in the destruction and degradation of cartilage in the pathogenesis of OA. Huang et al. confirmed that WNT5A can promote chondrocyte catabolic activity through a non-canonical Wnt signaling cascade in OA cartilage [148]. EV-associated miR-92a-3p can suppress the production of WNT5A, which is largely involved in the pathogenesis of OA. Increased expression levels of miR-92a-3p in EVs from MSCs and significantly decreased levels in EVs from OA chondrocytes were confirmed. Treatment with EVs containing miR-92a-3p increased chondrocyte proliferation by suppressing WNT5A protein production [67].

Similarly, miR-140-5p found in EVs from synovial MSCs has shown promising effects. The regulation of the Wnt signaling cascade by miR-140-5p in a rat model has been demonstrated to support cartilage tissue regeneration and protect against knee OA [68]. EVs from MSCs significantly reduced the expression levels of catabolic genes in IL-1β activated human chondrocytes. Analysis of miRNA content in these EVs revealed that these particles contain chondroprotective miR-140 and miR-451 [149]. It was confirmed that miR-26a-5p specifically binds to *PTGS2* (gene encoding prostaglandin-endoperoxide synthase 2). Overexpression of miR-26a-5p in EVs from bone marrow stem cells (BMSCs) has a regenerative effect on damaged synovial fibroblasts through suppression of *PTGS2* expression [69]. Wu et al. reported in their study that miR-100-5p in EVs isolated from IFP-MSCs protects articular cartilage from damage and regulates its homeostasis through inhibition of the mTOR-autophagy pathway [70]. In the future, isolated EVs from IFP might be used as potential biomarkers of OA. Another study confirmed that EVs can promote the proliferation of chondrocytes through miR-302c and inhibit the production of MMP-13, as the main degradative enzyme of cartilage ECM [150].

### 4.3. EV-Associated lncRNA Biomarkers

In addition to EV-associated miRNAs, lncRNAs have been reported to be involved in promoting proliferation and inhibiting apoptosis of chondrocytes during OA. The heterogeneity of lncRNAs is due to their size, which ranges from several hundred to several thousand nucleotides. They have an important regulatory role in the processes of development, differentiation, proliferation, apoptosis and cell metabolism. However, it seems that it is not their size, but the secondary and tertiary structures that are essential in the correct performance of the functions of these molecules [151].

Three groups of participants (the control group, the early OA group, and the late OA group) were used in a research by Zhao and Xu [71]. All individuals provided blood samples from the elbow vein and knee joint SF samples. EVs were isolated using ultracentrifugation, and RT-PCR was used to assess the expression of a number of EV-associated lncRNAs. EV-associated lncRNA PCGEM1 expression progressively rises as OA progresses. This demonstrated that EV-associated lncRNA could be a novel molecular biomarker for the precise and efficient monitoring of OA development. Kohle et al. found that the miRNA content of the EVs from SF differ between OA and non-OA groups. In males, 69 miRNAs were significantly downregulated and 45 miRNAs were upregulated. In females, 91 miRNAs were downregulated and 53 miRNAs were upregulated. The data showed gender-specific differences in miRNA content in OA EVs [152]. EV-associated lncRNA KLF3 AS1 from MSCs affects chondrocyte proliferation and inhibition of chondrocyte apoptosis through the miR-206/GIT1 pathway [72]. EV-associated lncRNA PVT1 regulated OA progression by modulating HMGB1/TLR4/NF-κB pathway through miR-93-5p [73]. The experimental results confirmed that the EV-associated lncRNA LYRM4-AS1 regulated the growth of activated chondrocytes through the GRPR/miR-6515-5p pathway and alleviated the inflammation present in OA [74]. Reviewing the roles of EVs containing miRNAs and lncRNAs and the research progress of these molecules can help to better understand the pathogenesis and to discover new molecular biomarkers of OA.

## 5. Conclusions

Refined diagnostic approaches need to be developed that identify OA subtypes and indicators of its progression, at earlier stages of the disease. This approach may enable personalized interventions that offer patients a better chance of preserving joint function and reducing pain. Success in this field has the potential to improve the lives of the many millions of OA sufferers worldwide. The ideal biomarker could be collected non-invasively, be predictive of the outcome of the disease and also provide potential therapeutic targets. The current markers all have advantages and disadvantages. Bodily fluids such as blood or urine are easily accessible, but it is known that changes in the synovial fluid can be detected earlier suggesting that markers may have a higher sensitivity and specificity in synovial fluid. MiRNA-based diagnosis nowadays represents the most dynamic area in OA biomarker research. Several studies have identified OA-specific miRNAs but there is a need to validate these results with a large-scale sample size in preclinical and clinical studies. For early and accurate diagnosis of OA, mainly knee OA, there is a need to identify complex panels of soluble biomarkers in various biofluids (serum, urine and synovial fluid) as well as in EVs to predict early-stage OA in a more precise manner. There are several options for measuring OA biomarkers, ranging from simple and relatively inexpensive methods to complex and demanding laboratory techniques. The development of tests should prioritize multiplex marker miniaturization and chip-based measurement in order to increase the cost-effectiveness of OA diagnostics and monitoring.

## Figures and Tables

**Table 1:** Potential OA biomarkers.

Type	Category	Molecule	Bodily Fluid/ MSCs Type	Ref.
Soluble	Inflammatory	IL-1β	plasma, SF	[44,45]
		TNFα	N/A	[46]
		IL-6	SF, serum	[47]
		CCL2	SF	[48]
		CCL3	serum	[49]
		CCL5	SF	[50]
		CCL13	SF, serum	[51]
		CXCL12	SF	[52]
	Collagenous	CTX-II	urine	[53,54,55]
		Coll 2-1	urine	[56]
		C2C	urine	[57]
	Non-collagenous	COMP	SF, serum	[58,59]
		S100	serum	[60]
EV-associated	Protein	IL-1β	SF	[61]
		IL-17	SF	[61]
		IL-10	SF	[61]
		INFγ	SF	[61]
		TNFα	plasma	[62]
		haptoglobin (female)	SF	[63]
		orosomucoid (female)	SF	[63]
		ceruloplasmin (female)	SF	[63]
		β-2-glycoprotein (male)	SF	[63]
		complement component 5 (male)	SF	[63]
	Lipid	phospholipid	SF	[64]
	miRNA	miR-95-5p	MSCs-EVs	[65]
		miR-193b-3p	plasma-EVs	[66]
		miR-92a-3p	MSCs-EVs	[67]
		miR-140-5p	MSCs-EVs	[68]
		miR-26a-5p	BMSCs-EVs	[69]
		miR-100-5p	IFP-MSCs-EVs	[70]
	lncRNA	PCGEM1	SF-EVs	[71]
		KLF3 AS1	MSCs-EVs	[72]
		PVT1	serum-EVs	[73]
		LYRM4-AS1	BMSCs-EVs	[74]

IL-1β: interleukin-1 beta; TNFα: tumor necrosis factor alpha; IL-6: interleukin 6; CCL2: monocyte chemoattractant protein-1; CCL3: macrophage inflammatory protein-1 alpha; CCL5: regulated upon activation, normal T cell expressed and secreted; CCL13: CC motif chemokine ligand 13; CXCL12: CXC motif chemokine ligand 12; CTX-II: C-terminal telopeptide of collagen type II; Coll 2-1: type II collagen-specific biomarker; C2C: type II collagen cleavage product; COMP: cartilage oligomeric matrix protein; S100: proteins of S100 family; IL-17: interleukin 17; IL-10: interleukin 10; INFγ: interferon gamma; PCGEM1: prostate-specific transcript 1; KLF3 AS1: KLF3 antisense RNA 1; PVT1: Pvt1 oncogene; LYRM4-AS1: LYRM4 antisense RNA 1; SF: synovial fluid; MSCs-EVs: mesenchymal stem cell-derived extracellular vesicles; BMSCs-EVs: bone marrow mesenchymal stem cell-derived extracellular vesicles; IFP-MSCs-EVs: extracellular vesicles from infrapatellar fat pad-derived mesenchymal stem cells; SF-EVs: synovial fluid-derived extracellular vesicles; serum-EVs: serum-derived extracellular vesicles; N/A: not available.

## Data Availability

Not applicable.
